# Investigating the stability of SuperAging over time

**DOI:** 10.1002/alz.091472

**Published:** 2025-01-03

**Authors:** Caitlin C Loxton, Tamara G. Fong, Richard N Jones, Edward R Marcantonio, Eva M Schmitt, Yuta Katsumi, Lisa F Barrett, Alexandra Touroutoglou, Bradford C. Dickerson, Sharon K. Inouye, Bonnie Wong

**Affiliations:** ^1^ Massachusetts General Hospital, Boston, MA USA; ^2^ Frontotemporal Disorders Unit, Massachusetts General Hospital, Boston, MA USA; ^3^ Harvard Medical School, Boston, MA USA; ^4^ Marcus Institute for Aging Research, Hebrew SeniorLife, Boston, MA USA; ^5^ Beth Israel Deaconess Medical Center, Boston, MA USA; ^6^ Alpert Medical School of Brown University, Providence, RI USA; ^7^ Northeastern University, Boston, MA USA; ^8^ Massachusetts Alzheimer’s Disease Research Center, Massachusetts General Hospital, Boston, MA USA

## Abstract

**Background:**

“SuperAgers” (SA) are older adults who perform significantly better than their peers and comparable to young adults on objective memory measures. Longitudinal studies show that many do not maintain their SA status over time. The fluctuation in SA stability may reflect changes in executive functioning, hypothesized to contribute to variance of episodic memory scores in SA. In the present study we analyzed longitudinal neuropsychological performance of SAs in the Selective Aging After Elective Surgery (SAGES) study to examine SA stability. We hypothesized that losing SA status over time will reflect a decline in executive function.

**Methods:**

17 SAs (criteria defined by Katsumi, Wong et al., 2022) (mean age 75.4, 12 females) identified in the SAGES Study were tested annually (up to 84 months). Neuropsychological data for baseline and follow‐up at years 1, 2, and 3 were analyzed. Performance of SAs who no longer met SA criteria after baseline (non‐stable SAs) were compared to that of SAs who maintained their status (stable SAs). A mixed factorial ANOVA was run with Trail Making Test B (TMTB) scores as the dependent variable to determine differences over time between stable and non‐stable SAs.

**Results:**

7/17 non‐stable SAs were identified. Analyses revealed a significant group x timepoint interaction [F(3, 39) = 3.152, p < 0.05]. Assessment of the coefficient of variation calculated across time points revealed that non‐stable SAs showed greater variability in TMB performance (M = 0.21, SD = 0.059) than stable SAs (M = 0.15, SD = 0.064). A significant difference was found between stable SA and non‐stable SA for TMT‐B performance [F(1, 13) = 13.237, p < 0.01. Both groups were similar in age at baseline (p < 0.19).

**Conclusions:**

These findings suggest that current SA definitions need refinement as SA reflects more than superior memory. Ongoing analyses include 7‐year data from the current sample, replication of findings in an independent SA cohort, and examination of the neuroanatomical correlates of the observed differences between stable and non‐stable SAs. Enhancing our understanding of SAs will deepen our knowledge of resilience to decline in aging.

